# Thermal imaging in biomedical research: a non-invasive technology for animal models

**DOI:** 10.3389/fvets.2025.1544112

**Published:** 2025-02-24

**Authors:** Antonio Verduzco-Mendoza, Adriana Olmos-Hernández, Antonio Bueno-Nava, Dina Villanueva-García, Adriana Domínguez-Oliva, Alberto Avila-Luna, Patricia Mora-Medina, Arturo Gálvez-Rosas, Ismael Hernández-Ávalos, Alejandro Casas-Alvarado, Marco A. Garnica, Daniel Mota-Rojas

**Affiliations:** ^1^Universidad Autónoma Metropolitana, Mexico City, Mexico; ^2^Department Bioterio and Experimental Surgery, Instituto Nacional de Rehabilitación-Luis Guillermo Ibarra Ibarra, Mexico City, Mexico; ^3^Division of Neurociences, Instituto Nacional de Rehabilitación-Luis Guillermo Ibarra Ibarra, Mexico City, Mexico; ^4^Division of Neonatology, Hospital Infantil de México Federico Gómez, Mexico City, Mexico; ^5^Neurophysiology, Behaviour and Animal Welfare Assessment, DPAA, Universidad Autónoma Metropolitana, Mexico City, Mexico; ^6^Facultad Estudios Superiores Cuautitlán, Universidad Nacional Autónoma de México, FESC, Cuautitlán, Mexico; ^7^Unidad de Agudos y Choque Adultos, Centro Nacional de Investigación y Atención de Quemados, Instituto Nacional de Rehabilitación Luis Guillermo Ibarra Ibarra, Mexico City, Mexico

**Keywords:** infrared thermography, burn injury, vascular surgery, flaps, grafts

## Abstract

Thermal imaging has been used in animal models to non-invasively detect surface temperature changes after several pathologic and surgical processes. Infrared thermography (IRT) identifies increases or decreases in radiated heat according to blood circulation and microcirculation. The present review aims to discuss the most relevant aspects of IRT applied in biomedical research as a noninvasive technique in animal models, highlighting its importance in a clinical setting and for translational medicine. IRT provides an alternative to evaluate vascular anomalies where blood flow is interrupted. In surgical processes such as anastomosis and reconstructive techniques (e.g., grafts and flaps), thermal imaging can assess the viability of tissues. In burn injuries, IRT can predict and identify the areas of ischemia-necrosis and inflammation. Nonetheless, although IRT is a potential alternative to use in both animal models and human patients, the use of IRT and other imaging techniques is encouraged.

## 1 Introduction

Animal models in biomedicine are essential to comprehend the biological and physiological basis of several medical fields such as biology, immunology, infectious diseases, oncology, genetics, and neurosciences (1, 2). The responses of animal models to different diseases, drugs, surgical processes, and immunizations are usually evaluated through cardiorespiratory, metabolic, endocrine, and behavioral parameters (3, 4). However, complementary and non-invasive techniques have been proposed in experimental research to avoid the stress-related response that invasive techniques might elicit in animals (5–7). An example is infrared thermography (IRT), a real-time and non-contact imaging technique that monitors body surface temperature (8–11).

IRT detects thermal radiation from the body surface with an accuracy of up to 0.1°C (10). The body’s surface temperature can change according to central and peripheral thermoregulatory mechanisms that result in vasoconstriction or vasodilation of superficial blood vessels to reduce or increase heat loss (12–14). Thus, it might robustly assess surface thermal variations in living organisms (15).

In mammals, physiological and pathological conditions (e.g., inflammation, fever, interruption of blood flow) induce alterations in thermoregulation mechanisms which can be assessed through IRT (15–19). Several authors have used IRT to map the thermal response in animal research, including rodent and porcine models (20, 21). For example, IRT has been used in vascular reconstruction models in rabbits, together with other advanced imaging techniques (e.g., angiography under ultrasonic observation) to describe the pathophysiology of thrombosis (22). In porcine models of intestinal and hepatic ischemia, IRT can detect circulatory changes and their association with tissue viability and blood flow restoration (23, 24). Similarly, in animal models of burn injuries, IRT can determine injury depth and predict the tissue healing (25).

**Figure 1:**
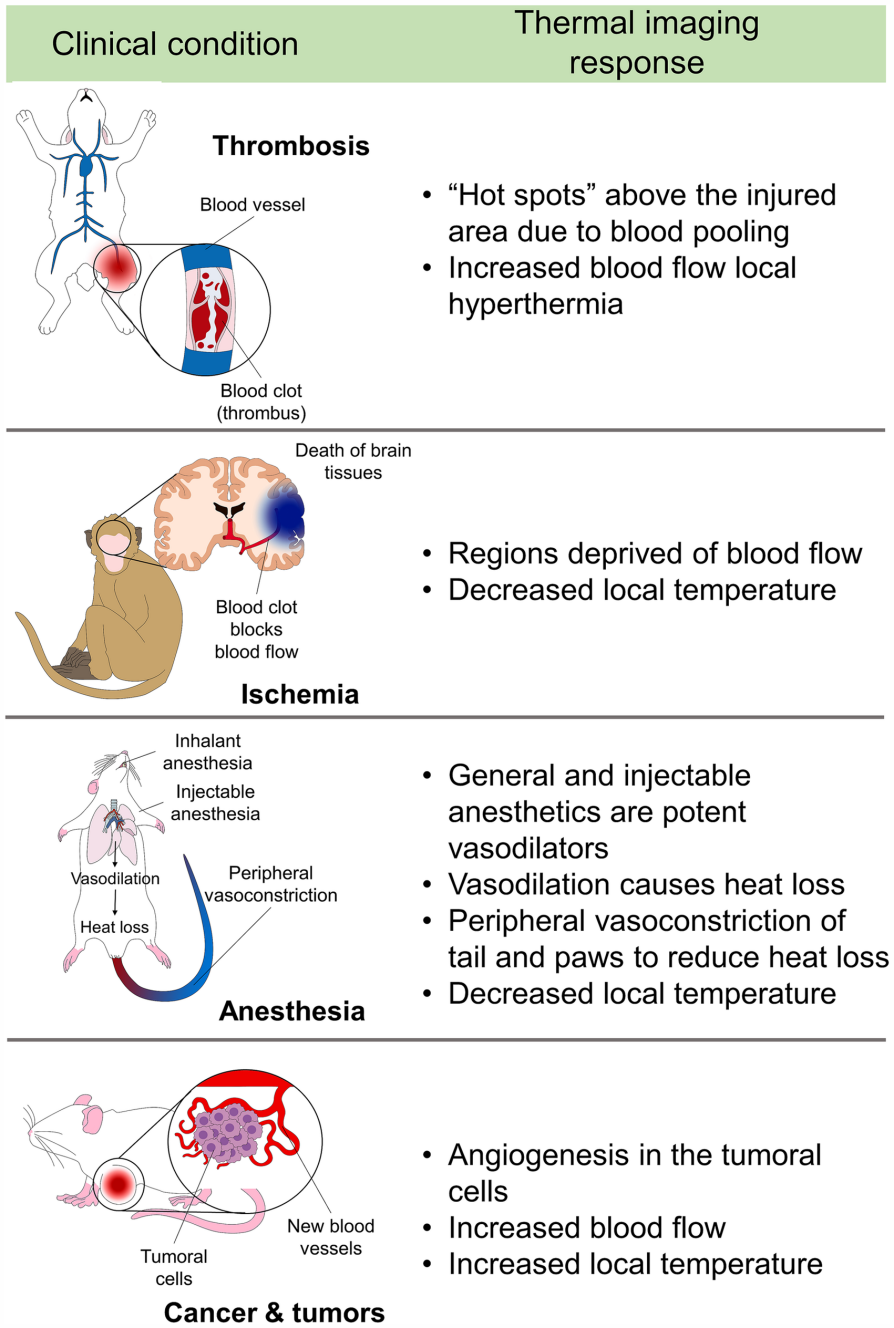
Summary of the application of thermal imaging in biomedical medicine and animal models.

Angiogenesis in oncological patients (e.g., breast cancer) is another aspect that has been replicated in murine models and studied with IRT. Processes such as hypervascularity and increased blood flow can be observed through IRT and are known as markers of tumor growth (Figure 1) (26, 27). Due to the wide field of research in which IRT is used in several animal models, this review aims to discuss the most relevant aspects of IRT applied in biomedical research as a non-invasive technique in animal models, highlighting its importance in a clinical setting and for translational medicine.

## 2 Search methodology

PubMed, Scopus, and Web of Science were the databases used for searching. The following keywords –or combination of keywords– were used to select relevant papers regarding “animal models,” “thermal imaging,” “diagnostic tool,” “experimental infrared thermography,” “thermal monitoring under anesthesia,” “burn injuries,” “vascular reconstruction,” and “blood flow restoration.” Included studies were those where thermal imaging was used as a complementary diagnostic tool in animal models of the mentioned clinical conditions or those that explain physiopathology. Likewise, papers using infrared thermography in both human and non-human patients were included. There was no settled date of publication, and all studies were written in English and Spanish.

## 3 Experimental vascular surgery, blood flow restoration, and its association with thermal imaging

**Figure 2:**
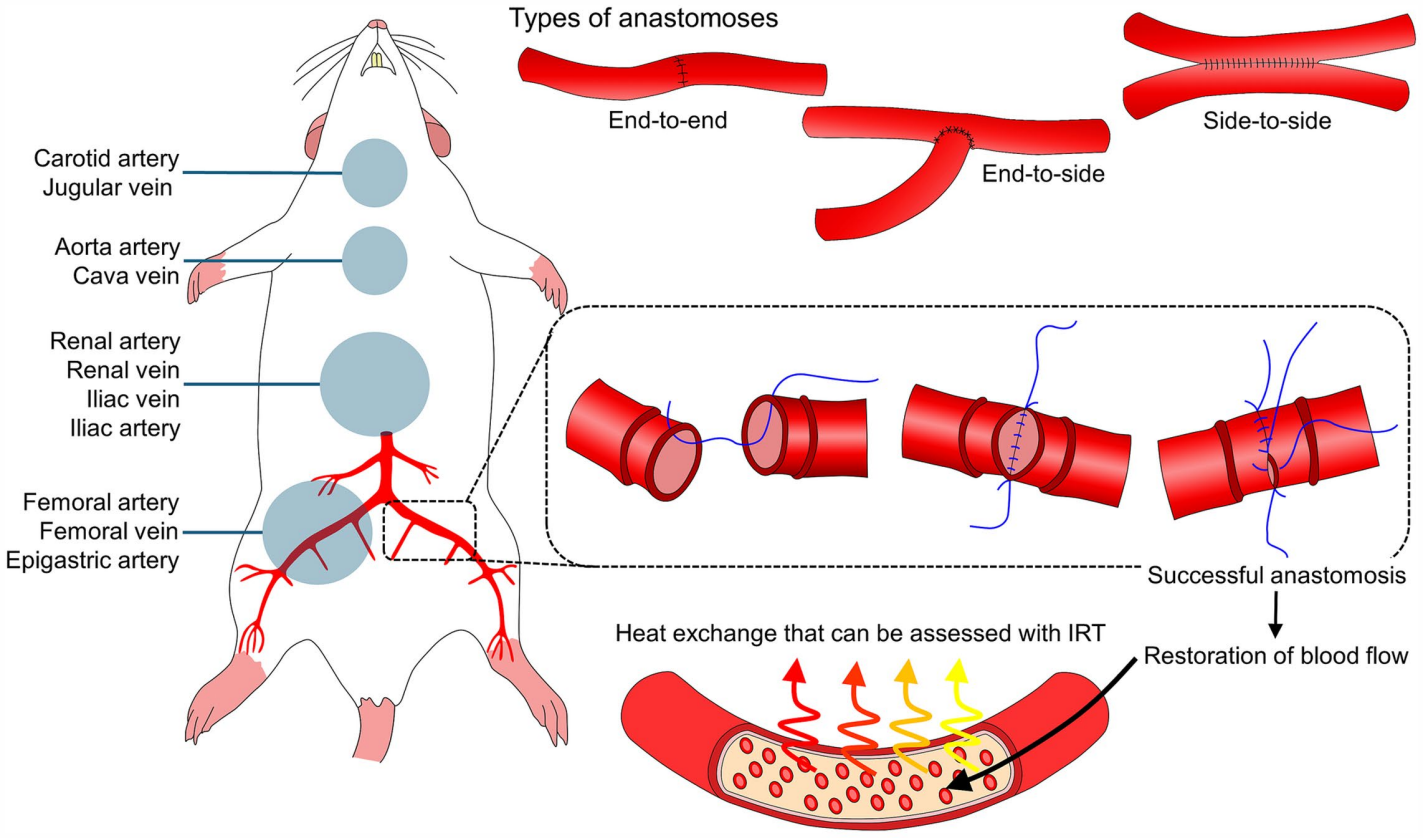
Anastomoses, animal models, and thermal imaging to monitor blood flow restoration. IRT: infrared thermography. In murine animal models, several vascular structures serve to practice and develop anastomoses techniques. Regardless of the type of anastomosis (e.g., end-to-end, end-to-side, and side-to-side), monitoring the restoration of blood flow after this type of vascular surgery is essential. After a successful anastomosis, restoration of blood flow elicits changes in the surface temperature of blood vessels and surrounding tissue.

Animal models such as small mammals and non-human primates are often used to study vascular pathologies including ischemia to understand the pathophysiological changes and associate them with injury degree and prognosis. In addition, surgical alternatives (e.g., anastomoses) are reproduced in animal models to evaluate their effectiveness (28). Vascular anomalies are followed by interruptions or blood flow deviations that alter the temperature of a tissue or organ. These changes can be assessed with IRT when an ischemic, thromboembolic, clamping, obliteration (obstruction) event, or another mechanism generates total or partial interruption of blood flow and, therefore, a reduction in the amount of radiated heat (Figure 2) (10, 29, 30).

### 3.1 Thrombosis and ischemia

Thromboembolism is one of the most common pathologies in peripheral blood vessels. Among these, venous thromboembolism is the most studied in human and animal models, in which IRT has been applied to identify changes generated by venous occlusion. An example is Deng et al. (22) study in rabbits, where thermal asymmetries were evaluated using IRT, ComPression UltraSonography (CPUS), and AngioGraphy Under ultrasonic Observation (AGUO) in a model of deep vein thrombosis in the femoral vein. The authors found that IRT identified an increase in the surface temperature of the affected right pelvic limb (hindlimb), ranging from 39.14–39.24°C to 41.08–41.91°C, suggesting that thermal imaging is a sensitive technique to detect changes in blood flow. Likewise, in humans by Bergqvist et al. (31), who compared IRT and conventional phlebography to identify deep femoral venous thrombosis. In 83.3% of cases, an increase in skin temperature was associated with thrombosis, and 90.1% of agreement was reported between the two diagnostic methods. Identification of a thrombus as a hot spot in a thermogram is due to blood pooling and increased blood flow to the region (12, 32).

In contrast to venous thromboses, the available literature on arterial occlusions is limited. However, these are considered of greater clinical interest due to the risk of severe complications such as limb loss, multi-organ dysfunction, premature intubation, stroke, and death (33). In vital organs such as the heart, Lekas et al. (34) have implemented IRT to identify the thermal changes of the epicardial surface in a canine model of temporary descending coronary artery occlusion. In the ischemic region, thermal imaging detected an immediate and progressive decrease from 31°C to 28–26°C. After reperfusion, the temperature returned to baseline values while some hypothermic and hyperthermic regions remained, representing the thermal imbalances that blood flow interruption can cause in the heart. In the same organ, Pouzot-Nevoret et al. (35) determined that IRT helps to differentiate feline aortic thromboembolism (FATE) with or without ischemia, due to the limb temperature decrease. In cats with acute bilateral pelvic paralysis due to FATE, the temperature of pelvic limbs was 22.7 ± 2.2°C, while in cats with acute hind limb paralysis not related to ischemic processes the average temperature of the limbs was 25.7°C. Moreover, in cats with FATE, the temperature of the pelvic limbs was 2.4°C below the one recorded in the thoracic limbs (unaffected region).

In the case of occlusions to the superior mesenteric artery, morphological and thermal imaging studies have been performed in Wistar rats, in whom ischemia was induced at five different times. Macroscopically, ischemia for less than 1.5 h caused moderate hyperemia, and the mesenteric surface temperature decreased by an average of 1.5°C. In contrast, rats subjected to prolonged ischemia (above 1.5 h) recorded a progressive decrease in temperature by up to 1.8°C. Subsequently, as a physiological response to reperfusion, short-term ischemia for 30 and 60 min increased the mesenteric temperature by 2°C to return to basal values. However, animals with prolonged ischemia did not return to basal values and maintained lower temperatures (36).

Organ ischemia has also been replicated in animal models, and IRT has shown certain applications to detect interrupted blood flow. Brooks et al. (23) compared IRT with conventional methods such as histology, Doppler ultrasound, and fluorescence to analyze intestinal ischemic damage induced in a porcine model. After mesentery ligation, IRT detected circulatory changes and established a percentage of nonviable tissue of 69.5%. This percentage was below the one obtained with fluorescence and Doppler ultrasound fluorescence and Doppler (91.8 and 80.8%, respectively). Thus, the authors concluded that IRT is recommended as a complementary alternative that must be used with other techniques to increase the accuracy of the evaluation. Likewise, induced hepatic injury by ischemia–reperfusion of the portal vein and left lobe hepatic occlusion in pigs has been evaluated with IRT by Gringeri et al. (24). Through IRT, the temperature of the ischemic lobes was found to be significantly lower than that of the non-ischemic right lobes (*p* > 0.05), and although rewarming was observed upon removal of the occlusion, hypothermia continued after 2 h. The authors highlighted the usefulness of IRT as a tool during the intraoperative period to detect early blood flow deprivation and its possible consequences such as hypoxia, the release of oxygen-free radicals, cytokines, microcirculatory failure, oxidative stress, and cell death (37, 38).

These findings are essential as they show that IRT can be used to identify revascularization (reperfusion) events, prevent ischemia, hypovolemia, and subsequent tissue necrosis in transplant surgeries, intestinal anastomosis, and other procedures where resection of the affected tissue must be decided. Likewise, IRT allows visualization of functional recovery after an ischemic process, where an increase in surface temperature is equivalent to a return of blood flow.

### 3.2 Anastomosis and blood flow restoration

Animal models have been used to perform microsurgical procedures such as the reconnection of blood vessels or vascular anastomosis. After this surgical procedure, clinical evaluation of the blood flow restoration is essential to establish the success and recovery of the patient. The high risk of failure after vascular anastomoses justifies the need to use evaluation methods in experimental animal models. Monitoring with IRT in the intraoperative period of vascular surgery could reveal some errors in the procedure and promptly establish correcting measures to guarantee the viability of the tissue (39, 40).

An example is Esposito et al. (40) study, which used IRT to compare conventional sutures with laser welding in bypass surgery in rabbits undergoing microvascular anastomosis. When evaluating the temperature of the region, as well as that of the edges of the carotid artery corresponding to the end-to-side anastomosis, the results showed that the temperature during and after the use of the laser did not interfere with the evolution of vascular restoration and there were no setbacks such as clot formation, equivalent to a successful anastomosis without leak points. Similarly, in a murine model of island pedicle flap, Li et al. (41) evaluated the changes in the surface temperature of the flap to determine the optimal delay periods for flap placement. A continuous white hotspot was observed in animals with a successful anastomotic vessel. In contrast, choke vessels had a visible red zone, showing the differences in temperature in both regions.

In humans with Moyamoya syndrome (carotid stenosis), IRT had a similar degree of diagnostic accuracy as conventional and validated imaging techniques (e.g., fluorescent marker indocyanine green) to evaluate anastomotic permeability during surgery to place a bypass (42). These studies suggest the application of IRT to detect intraoperative decreases in the temperature of injured organs that indicate decreased or lack of blood perfusion, which can help determine the extent of the damage and the prognosis of the affected tissue (24, 36). On the contrary, detecting increases or returns of surface temperature in anastomoses of blood vessels or organs ensures the success of the technique and prevents tissue loss that would lead to hypoperfusion (43).

### 3.3 Blood flow and tissue repair

The degree of damage and the healing process after a tissue injury (e.g., surgical wounds) are other fields where IRT could offer a non-invasive and complementary diagnosis (44–46). After tissue damage, a transient vasoconstriction of surrounding blood vessels is observed. In contrast, the inflammatory phase occurs 2–5 days after the injury, together with other tissue repair events such as angiogenesis and re-epithelialization (47–49). This effect has been observed when comparing the surface temperature of an injured and healthy skin zone, where the highest temperatures are recorded in the injured area due to the local inflammatory response that promotes tissue restoration (45). Indeed, high temperatures of up to 33.8 ± 0.9°C were associated with normal wound healing in human patients with thoracic surgical incisions. In comparison, values of 30.0 ± 1.2°C were observed in patients with local infectious processes (46). Moreover, this study reported that thermal imaging had a sensitivity and specificity of 91.6 and 85.7% in predicting healing status (46).

In the case of animal models, Deveci et al. (50) used IRT to monitor healing in a full-thickness skin wound model in Wistar rats. When comparing the local temperatures of untreated and treated (with dexpanthenol) animals, the authors found that as wound treatment time progressed, there was less local temperature increase. Similarly, in full-thickness cutaneous wounds in mice, treatment with europium dressing decreased the wound area faster than in control animals (51). Other options for comparing surgical procedures were reported by Viscardi et al. (52), who tested CO_2_ surgical laser as a refinement method to castrate piglets while improving the healing process of conventional procedures. Contrary to what was expected, laser-castrated piglets recorded lower wound temperatures than scalped-castrated piglets. Moreover, laser-castrated piglets had behavioral alterations related to pain, which suggested higher tissue damage than conventional castration techniques. These studies show that IRT can monitor the inflammatory response of the wound site and associate it with normal or delayed healing. Using IRT with other diagnostic tools might help provide an accurate healing prediction or even design intervention plans when wound healing is impaired.

## 4 Thermal imaging applied to neurosurgery and neurosciences

### 4.1 Experimental models of cerebral ischemia and neurosurgery

Neuroscience research includes neurosurgery, cerebral damage of different natures, and behavioral alterations, among others. Damage to nervous tissue, as well as its vasculature, can generate brain temperature alterations due to inflammatory, infectious, surgical, tumoral, or structural processes, events that have been recognized with thermal imaging (10, 53). For example, in a murine model of occlusion/reperfusion of the middle cerebral artery in hypertensive rats, Yao et al. (54), used IRT to non-invasively monitor brain temperature and maintain central brain temperature within a range of 1°C upper shift, serving as a support technology to conventional measurement methods through thermocouple probes.

Animal models are beneficial to study ischemic and occlusive processes in the middle cerebral artery, a vascular structure that irrigates both cerebral hemispheres and is exposed in surgical interventions (55). Therefore, structural damage such as temporary occlusion can cause thermal alterations, as demonstrated by Watson et al. (56) in a model of temporary occlusion of the branches of the middle cerebral artery and the internal carotid artery, in the region of the frontal, parietal and temporal lobes in the cynomolgus monkey (*Macaca fascicularis*). The regions deprived of blood flow recorded an immediate temperature decrease between 0.3 and 1.3°C, while they recovered basal temperatures after reperfusion. Likewise, the temperature of the arterial blood vessels decreased between 1.3 to 3.2°C.

The sequelae of vascular events have also been studied in humans but using IRT, as reported by Alfieri et al. (57) in patients with hemorrhagic stroke. The authors found that, when compared to the control group, the temperature of the hands in individuals who suffered unilateral hemiparesis after stroke had similar values (29.5 ± 3.0°C vs. 29.6 ± 2.7°C). In contrast, significant differences were detected in the feet of affected (27.5 ± 3.3°C) versus healthy patients (27.9 ± 3.0°C). However, da Silva Dias et al. (58) did not find differences in foot temperature between patients with loss of somatosensory sensitivity and those with stroke sequelae.

Neurosurgery and the perioperative period, including the surgical procedure and anesthesia, is another field where IRT has shown its clinical application. In the case of anesthesia monitoring, Kastek et al. (59) evaluated the effect of anesthetics such as isoflurane and the changes in cerebral flow due to the effect of CO_2_ and occlusion of the right middle cerebral artery. The authors reported that 80% of arterial occlusion decreases brain surface temperature by approximately 0.5°C below the baseline value. This effect was reversed after reperfusion, recording increases of up to 1.6°C, as a result of a sudden influx of blood to the brain. Likewise, Suzuki et al. (60) evaluated cerebral metabolism and blood flow with IRT using isoflurane and *α*-chloralose. Isoflurane increased cerebral circulation but decreased metabolism, in contrast to α-chloralose, showing the application of IRT to detect circulatory alterations and the consequent changes in radiate heat.

**Figure 3:**
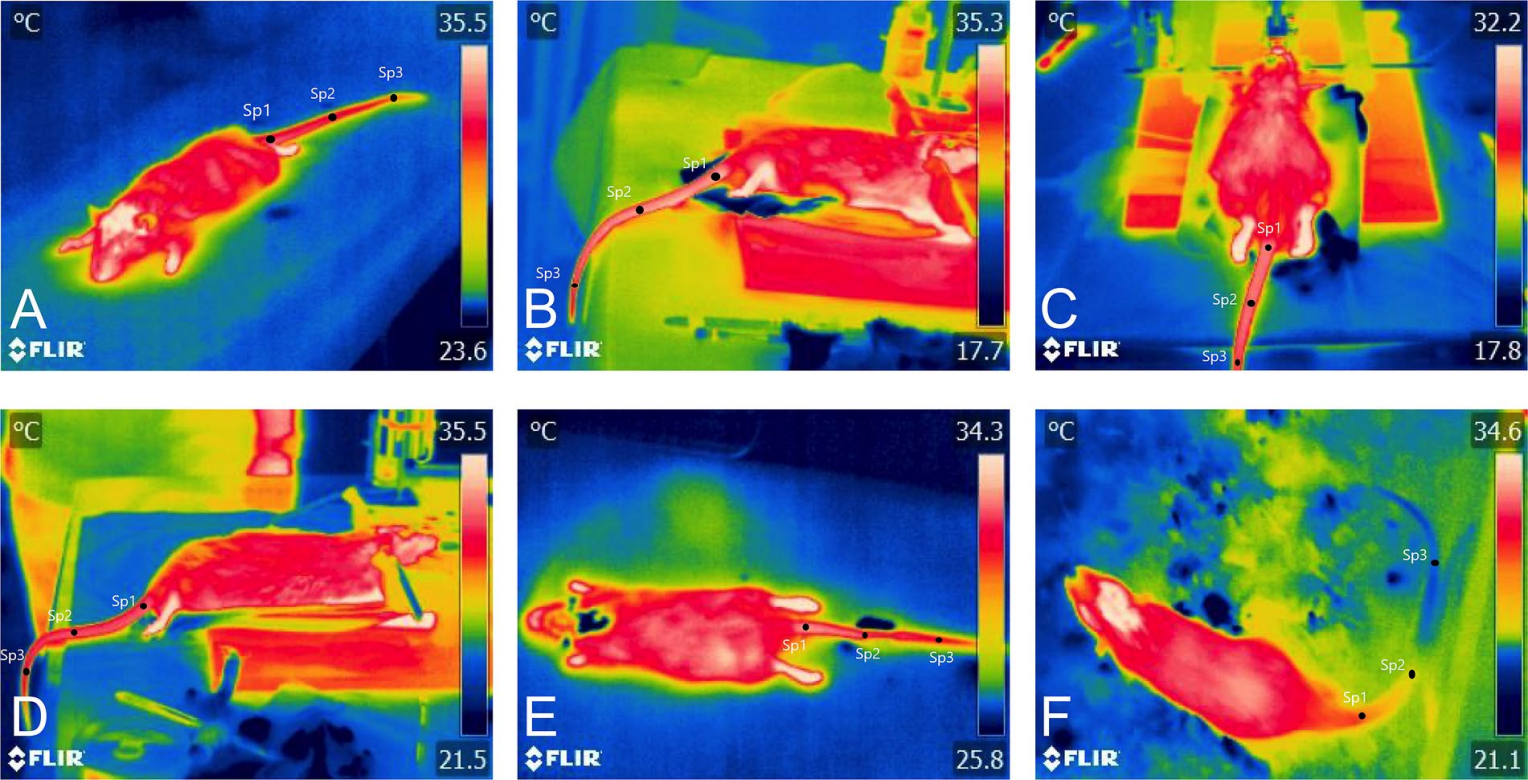
Thermal images of a brain injury model in Wistar rats. During stereotactic surgery, thermal imaging was performed to record the changes in the average temperature of the tail, the main thermoregulatory organ of rats. **(A)** Before the skin incision, it can be observed that the tail temperature of the proximal (Sp1), medial (Sp2), and distal segments (Sp3) was within a range of 32.5 and 31.1°C. **(B)** Incision. In contrast to the basal period, the tail temperature dropped by up to 1.9°C in the proximal segment of the tail. This could be associated with the effect of anesthetics, which usually cause hypothermia after their administration. **(C)** Trephine hole. A progressive decrease in temperature was observed in the animals, dropping up to 3.5°C compared to the temperatures before the incision. **(D)** Induced brain injury. Increases in tail temperature were recorded in all three segments (32.4°C, 32.3°C, and 32.0°C). Because head trauma can be considered a stressor that also causes pain, vasodilation in the tail could be associated with surgical stress-induced hyperthermia. **(E)** Suturing. Small decreases in temperature were recorded during suturing, with values of −0.7°C, −0.2°C, and − 0.3°C in the proximal, medial, and distal segments, respectively. **(F)** Post-injury. Similar to suturing, decreases in tail temperature of up to 6.8°C were recorded. The decrease in temperature may be related to the vasodilatory effect of anesthetics that promotes heat loss and, therefore, a lower amount of heat radiated to the environment.

The thermal images included in Figure 3 show another suggested application of IRT during an experimental process such as stereotactic surgery in a rat model. With IRT, the temperature changes from the perioperative period can be observed. For example, during anesthesia induction, the temperature of the tail (one of the main thermoregulatory organs of the species) decreased. In contrast, an incision generates pain or surgical stress, with an increase in physiological parameters, something similar to what can be observed during the induced brain injury model, that is likely to trigger an adrenergic discharge. All these events induce immediate physiological changes that can be recorded with IRT. These results suggest that IRT could be used during invasive neurosurgical procedures and/or those that require high-precision manipulation (aneurysms, arteriovenous malformations, or tumors), due to the irrigation of these highly vascularized structures. In this sense, IRT is useful to determine active areas on the exposed cerebral cortex, due to the increase in blood flow and consequently metabolism during motor and language tests in the trans-operative period (61). Similarly, Parrish and Iorga (62) used IRT to monitor brain function in human patients undergoing glioma resection surgery. The results showed that intraoperative thermography detected changes in the thermal pattern before and during the behavioral task, showing the brain regions that are activated when an action is requested. For example, when the patient was asked to squeeze an object with his right hand, the temperature of the sensory and motor cortex increased due to increased vascularization.

### 4.2 Infrared thermography and animal models in behavioral neuroscience

Several induced behavioral responses in animal models are another field where IRT has been used due to the association of peripheral blood flow control and central responses. Blood flow depends on the autonomic control that innervates the blood vessels. Thus, superficial or cutaneous thermal changes might be associated with fear and other emotions as shown by Vianna and Carrive (63) in Wistar rats. In this study, after a conditioned fear test, the temperature of the tail (31.5°C) and the foot (34.8°C) decreased by an average of 5.3°C and 7.5°C, respectively. In comparison, the eye temperature (35.2°C) and the head temperature (32.8°C) increased by an average of 0.8°C and 1.5°C, respectively (63). This response is the result of the endocrine responses elicited by stress. The vasomotor effects reduce the temperature in peripheral areas (such as the tail and legs) and increase the temperature in key organs (e.g., eyes and brain) to cope with the stressful stimulus (64). During stress, the hypothalamic–pituitary–adrenal axis is activated, stimulating the adrenal cortex and the secretion of glucocorticoids, which promote gluconeogenesis and lipolysis, which increases thermogenesis. In turn, glutamatergic neurons stimulate the dorsomedial hypothalamus, which promotes sympathetic activation and thermogenesis, generating stress hyperthermia (65).

Other studies have also reported increases in head/body temperature and decreases in tail surface temperature in mice in response to stress caused by movement restriction (66, 67). Lecorps et al. (68) measured the thermal response with IRT on the tail and body surface of mice exposed to fox feces (predator odor). The mice responded with a discrete increase in tail temperature, from 22.10 ± 0.9°C to 23.32 ± 1.3°C. In animal models of anxiety, Miyazono et al. (69) studied the anxiolytic effect of etizolam in a stress-fear model caused by mice exposed to pyrazine, a molecule that has been shown to generate fear in rats and mice. Using IRT, it was observed that pyrazine prevented significant decreases in tail temperature, recording decreases of less than 1°C. Likewise, Lecorps et al. (70) indicated that in the elevate plus maze and open-field neuropsychological tests, the infrared eye temperature increased (*R* = 0.213), while the tail temperature decreased, both as the test execution time passed. This means that animals subjected to this type of neuropsychological test generate a certain level of stress and/or anxiety, determined by the relationship that exists between the increase in temperature during the test. It should be noted that the non-invasiveness achieved with thermography allows these findings to be obtained, aspects that must be considered by researchers when issuing their conclusions.

**Figure 4:**
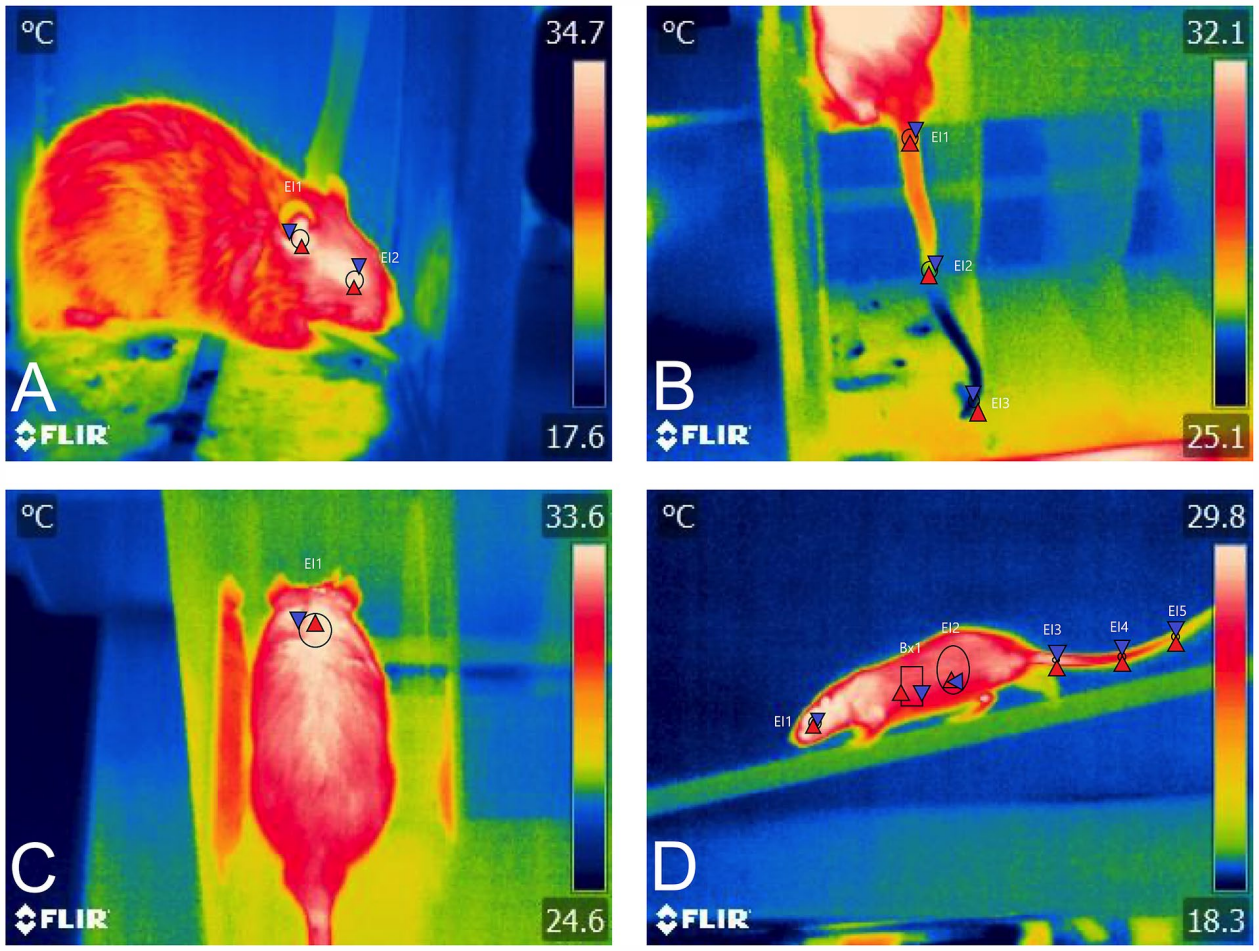
Application of IRT in various neurobehavioral tests. **(A)** During a conditioning protocol to electroshocks, the average eye temperature (El1) and the ear (El2) can be evaluated with IRT, recording 34.7°C and 35.4°C. **(B)** Administration of the dopaminergic drug SKF. The vasoconstriction effect of the drug can be assessed by recording the surface temperature of the tail, obtaining minimum temperatures of 25.5°C at the distal end of the tail (El3). **(C)** During the Rotarod motor test, the increase in temperature in the BAT region (El1) is identified, which may be associated not only with locomotion but with the degree of stress during the test. **(D)** Motor test in an elevated beam paradigm. A lateral view of the rat during its displacement could provide information on the thermal response of the animals.

Other studies focused on stress, such as that of Carrive et al. (71), reported a reduction in pain sensitivity in rats conditioned to stress, which was called “stress-induced analgesia.” The decrease in tail temperature (4.4°C) recorded with IRT during stress was correlated with the slowing of nerve fiber conduction. On the other hand, Faraji and Metz (15) used IRT to demonstrate that female mice tend to have greater temperature modification when compared to males in stressful situations. In a rearing deprivation test, the skin temperature of the head of females was 33.75°C, while males recorded 33.40°C. Similarly, the temperature of the female’s back was 33.33°C versus 32.97°C of males. Regarding tail temperature, the values in females were an average of 27.21°C while the males recorded 27.91°C. Thus, IRT could be used as a tool to recognize surface temperature changes elicited by stress-mediated responses, as proposed in Figure 4.

## 5 IRT and anesthesia monitoring in laboratory animals

One of the main challenges during anesthesia is maintaining thermal homeostasis due to the tendency of patients to develop hypothermia during the perioperative period. During a surgical procedure, shaving, application of antiseptics on the skin, and organ exposure increase body heat loss by radiation and evaporation. Moreover, due to the depressing effect of anesthetics on the central nervous system, they decrease the sensitivity of the hypothalamus -the main thermoregulatory center- to detect low temperatures, resulting in an inhibition of the compensatory response to raised temperature (e.g., thermogenesis and sympathetic inhibition) (72). Due to the reported thermal changes, IRT has been used to evaluate the regional temperature in subjects with local anesthetic effects, mainly spinal cord blocks and general anesthesia.

### 5.1 Local anesthesia and surface thermal changes

The effect of local anesthetics has been studied mainly in murine animal models to understand their mechanism of action or to corroborate the technique’s efficacy. In this sense, Xu et al. (73) evaluated the surface temperature of the fore and pelvic limbs, ears, and tail of C57BL/6 mice receiving 50-μL bupivacaine 0.25% epidurally. The temperature of the tail and pelvic limbs progressively increased by approximately 3 to 4°C. The increase in temperature in these structures is associated with the vasodilation caused by the local anesthetic, increasing blood flow to the caudal region. This thermal effect is the result of the suppression of neurological activity due to the regional epidural block. Thus, increases in the regional temperature after the administration of a local anesthetic might help to confirm the success of the epidural block. Another study in rats evaluated the effects of the administration of ropivacaine (local anesthetic) by recording the temperature of the head, back, and tail. The authors found that the animals that received the drug had a similar maximum temperature in the head (central compartment) and the back (peripheral compartment) (29.4°C and 30°C, respectively). The animals also presented microcirculatory changes in the tail that caused congestion and therefore an increase in caudal temperature (74).

In canines, Küls et al. (75) recorded the temperature of the pad region before orthopedic surgery in dogs to evaluate the effect of epidural and femoral-sciatic blockade with bupivacaine. With IRT, it was determined that a temperature difference greater than 1°C was associated with a successful anesthetic block. In the same species, Wan-Tae et al. (76) evaluated the temperature of the dorsal region of dogs with spinal cord injury by experimental compression using balloon inflation (Foley catheter) at the L2 and L3 levels. The findings obtained by IRT reported a decrease in temperature from the thoracic region to the pelvic level, recording the lowest temperatures in the pelvic region. In these animals, in the fourth week after surgery, a slight increase in temperature was observed, mainly in the thoracic region. Likewise, it was found that canines maintained dorsal temperature symmetry before and after surgery, where the lateral temperature of the left thoracic, lumbar, and pelvic region was 28.66, 28.48, and 28.09°C, respectively; and those of the right side were 28.65, 28.47, and 28.09°C, respectively. Therefore, IRT can assess the temperature changes as a response to regional anesthesia, where a successful blockade is interpreted with a decrease in regional temperature.

### 5.2 General anesthesia and its effect on peripheral thermoregulation

General anesthetics (both inhalational and parenteral drugs) induce hypothermia, a state that can be evaluated with IRT, as shown by Gjendal et al. (67) who assessed the thermal response of C57BL/6 mice to 3 stimuli; (1) anesthesia with isoflurane, (2) scruff, and (3) injection of substances intraperitoneally. This study showed that isoflurane induced a tail temperature drop by more than 3°C during anesthesia (basal: 31.16°C *vs*. anesthetic: 28.13°C), followed by a drop in eye temperature (from 37.32°C to 34.70°C) and body temperature (from 32.88°C to 30.29°C). Vogel et al. (77) mention that ocular surface temperature is a good indicator to measure the effect of isoflurane-induced anesthesia in an animal model of myocardial infarction, while Hankenson et al. (72) describe that isoflurane anesthesia causes dilation of peripheral blood vessels, exacerbating heat elimination, especially in the thermoregulatory organ of rats, which is the tail.

In other studies where the tail has been used as a thermal window to evaluate the effect of injectable anesthetics such as intraperitoneal ketamine (150 mg/kg), anesthetized C57BL/6 mice showed an immediate decrease in tail temperature, a change that persisted at 5, 10, 15 min and recovery (32.35 ± 1.60°C), 5 (27.23 ± 0.51°C), 10 (25.19 ± 0.52°C), (25.18 ± 0.89°C), (25.39 ± 0.80°C), respectively. This hypothermia effect is associated with the mechanism of action as an N-methyl-D-aspartate receptor antagonist (78). Together with the hypothalamic suppression, general anesthetics inhibit the activation of *β*-adrenoceptors involved in the sympathetic response, which leads to a decrease in heart and respiratory rate, as well as the inhibition of catecholamine release and the stimulation of BAT, responsible for non-shivering thermogenesis. In other species such as pigs, Farrar et al. (79) compared the rectal temperature and IRT values at the base of the ear and the temporal angle of eyelids in sows anesthetized with tiletamine and zolazepam. The results showed that the temperature of the base of the ear had a higher correlation with the rectal temperature (*r* = 0.432) than the ocular temperature (*r* = 0.359). Although this study showed that rectal temperature had less variability in terms of measurements, IRT is a tool that, due to its non-invasiveness, is an alternative for monitoring temperature in this species, in addition to the fact that other authors have reported strong positive correlations between rectal temperature and the ear canal (*r* = 0.62) and between rectal temperature and of the eye (*r* = 0.53) (80).

**Figure 5:**
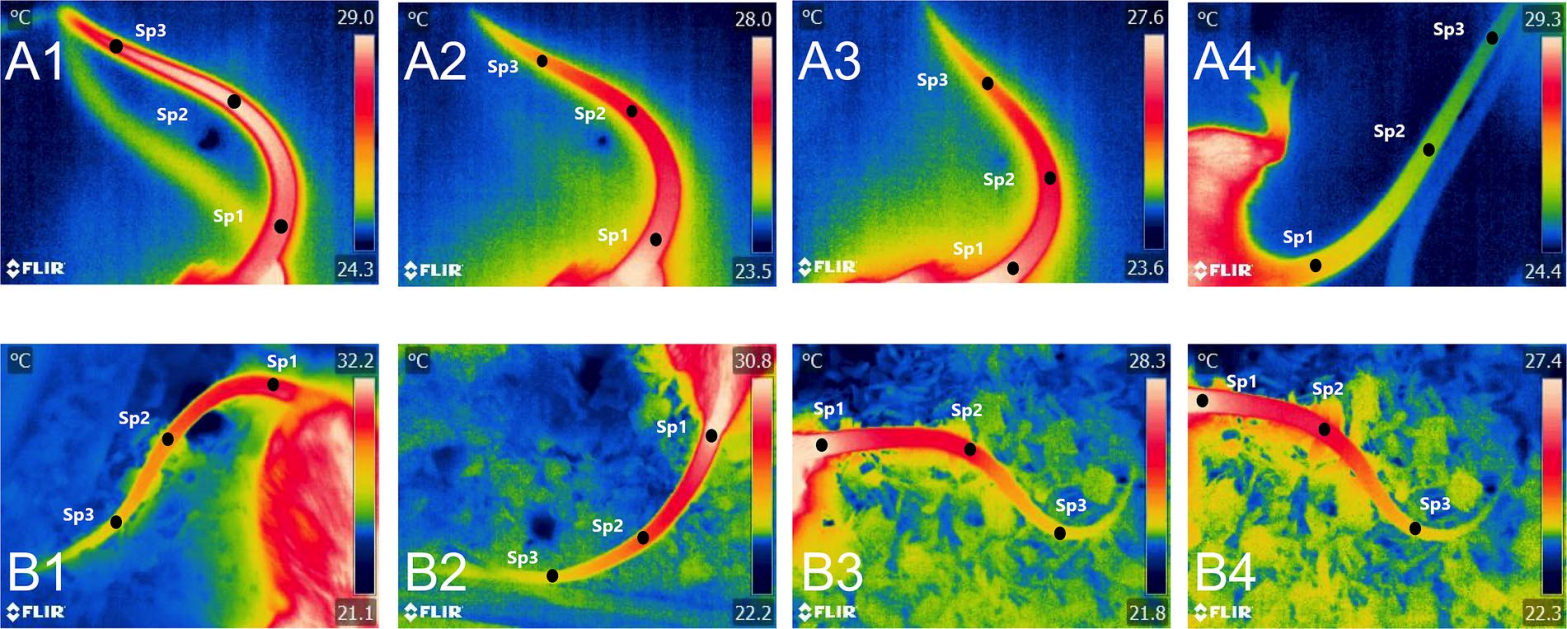
Thermal imaging was applied to evaluate inhalant and injectable anesthesia in female Wistar rats. Due to the influence of anesthetics on central thermoregulatory centers, IRT can be used to monitor the thermal balance of rodents during anesthesia. **(A)** Isoflurane anesthesia. A1 to A4 shows the progressive decrease in the temperature of the rat’s tail. When considering a proximal (Sp1), medial (Sp2), and distal segment (Sp3) of the tail, an average decrease of up to 3.3°C was registered. **(B)** Administration of ketamine + xylazine anesthesia. From B1 to B4, the injectable combination caused a progressive decrease in the temperature of the tail of up to 3.5°C. The decrease in the Surface temperature of the tail can be associated with the systemic vasodilator effect of general anesthetics that forces the organism to shift blood Flow from peripheral tissues to vital organs (e.g., heart and brain). Therefore, the surface temperature of the tail decreases due to vasoconstriction to reduce the amount of heat loss.

In this way, IRT could help to recognize and prevent complications during anesthesia, such as hypothermia. Hypothermia during anesthesia delays the recovery of consciousness and causes peripheral vasoconstriction (72). In animal models, the effect of anesthetics can be monitored through the surface temperature of the tail, which serves as a thermoregulatory organ. In this species, the decrease in temperature in the tail region is a physiological vasoconstriction process to preserve heat. This effect is schematized in Figure 5, where thermal imaging was used to monitor anesthesia in Wistar rats.

## 6 Thermal imaging to diagnose and monitor oncologic animal models

In humans, breast cancer affects approximately 2.8 million women worldwide. Although mammography is the most common technique for cancer diagnosis, other tools such as IRT are considered as an alternative due to the changes in temperature and increased blood flow as a result of possible angiogenesis (81). Angiogenesis is the formation of new blood vessels from existing capillaries. “Tumor angiogenesis” is the growth of new blood vessels that tumors need to expand. In the context of cancer, angiogenesis is a fundamental process for tumor growth and the formation of metastases (82). Cancer stem cells are also involved in neovascularization, and the presence of these cells has been found to contribute to the failure of various anticancer therapies through the process known as vascular mimicry formation (83).

The formation of new blood vessels in the tumor cell microenvironment is one of the reasons why IRT has been used in animal models of breast cancer. Through tumor growth monitoring, it was found that, unlike what is observed in humans with cancer, where the surface dermal temperature increases by 1–2°C, in mice, a progressive decrease in temperature was recorded (between 1.5 and 3°C, depending on the tumor cell) (26). In Sprague–Dawley rats, Wahab et al. (84) used thermography (together with four different image filtering techniques) for tumor recognition. The results showed that IRT not only allows the visualization of thermal changes in the tumor region, but its use in conjunction with other techniques (such as contrast stretching) improves the accuracy of IRT. In Balb/c mice, IRT was used to estimate tumor area according to changes in surface temperature after radiotherapy treatment, obtaining a maximum of 36°C on day 15, and a minimum of approximately 28°C on day 29. In addition, the results of IRT were compared with conventional measurement methods using manual caliper, finding a strong correlation between both techniques (85).

Other correlations between tumor size and tumor temperature have been reported in Balb/c mice. The temperature at the injection site progressively decreased from 33.7°C to 31.4°C, allowing temperature changes to be observed before clinical signs of the tumor appeared (86). Moreover, even when tumors cannot be diagnosed by direct observation, thermal imaging can detect temperature changes of up to 0.1°C in affected tissue (27).

Progressive decreases in surface temperature as the tumor increases in size are associated with necrosis in the center of the tumor due to the treatment (87). Therefore, the application of IRT in cancerology has not been limited to being a complementary diagnostic method but has also been suggested as a technique to monitor treatment, as studied by Yu et al. (88) in Sprague Dawley rats. In this study, IRT was applied to diagnose breast cancer and determine the effectiveness of treatment with doxorubicin nanoparticles (2 mg/kg). It was found that lower temperature was recorded in treated animals due to the efficacy of the treatment. Real-time IRT techniques in mice with subcutaneous tumors have been used with magnetic nanoparticle hyperthermia to approximate intratumoral temperature. In this sense, after 3 min of magnetic hyperthermia, Rodrigues et al. (89) used a formula developed for Andrä et al. (90) to distinguish tumor temperature from baseline values, which revealed significant differences between the surface temperature of healthy mice (+11°K) and those with tumors (+5°K).

These studies in animal models have encouraged the application of IRT in humans, together with new precision medicine techniques. New technologies such as Deep Learning, when used together with IRT, have been shown to detect anomalies in thermograms of patients with breast cancer with a sensitivity of 92.3% and a specificity of 53.8% (91). Similarly, 100% accuracy has been reported by Fernández-Ovies et al. (92) when using IRT together with mammography and ultrasound. Thus, vascular changes characteristic of tumors, even without the presence of observable clinical signs, make IRT a valuable tool for the diagnosis and monitoring of anticancer therapy both in experimental models and in clinical trials with humans.

## 7 Animal models of burn injury and IRT

**Figure 6:**
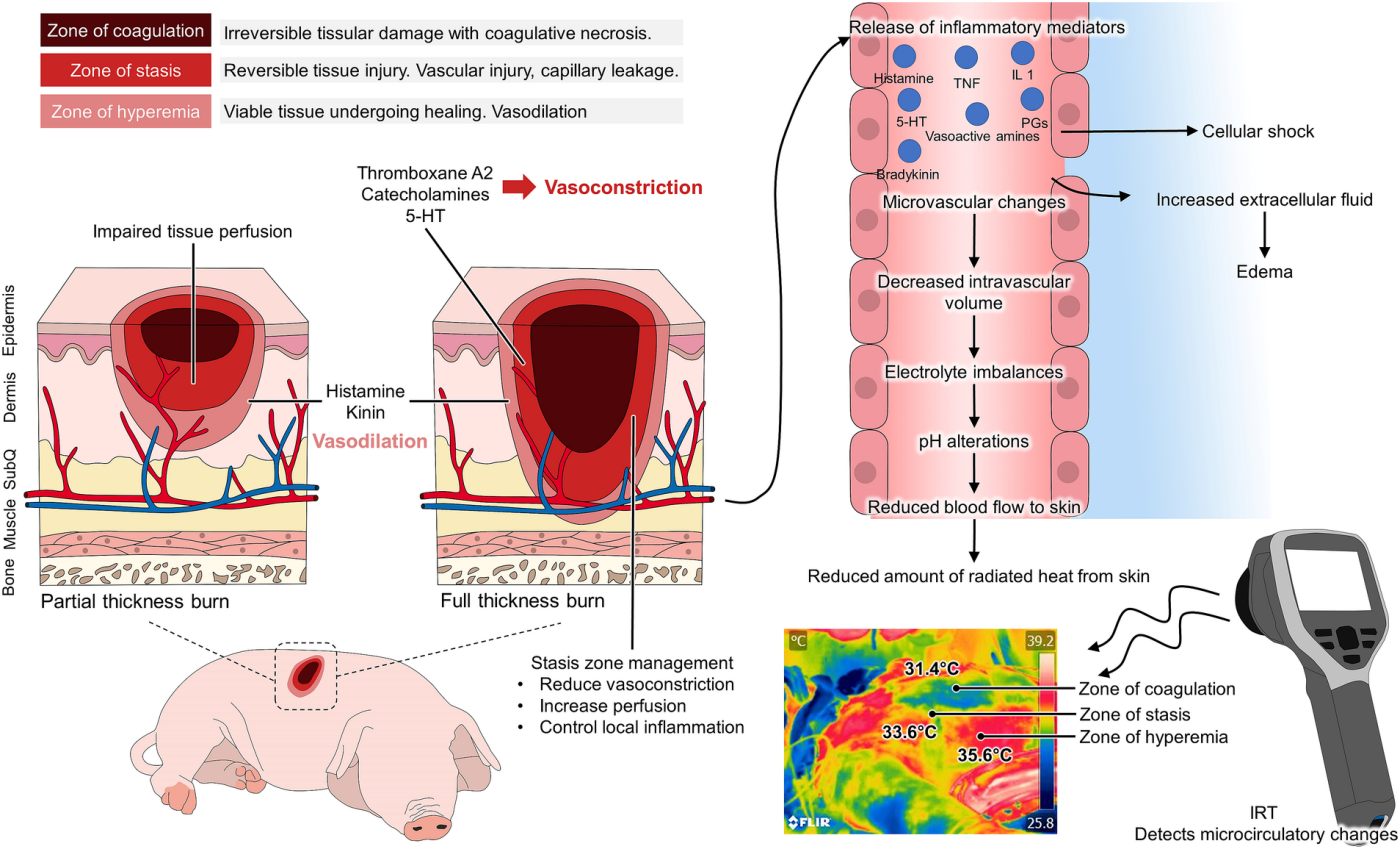
Application of thermal imaging in animal models of burn injury. According to the three burn injury zones, changes in the microcirculation arise after tissular damage. In the zone of coagulation, necrosis, and irreversible tissue damage are present. In the zone of stasis, reversible vascular injury is observed, causing vasoconstriction due to the presence of chemical mediators such as thromboxane and catecholamines. In contrast, in the hyperemia zone, vasodilation is present due to the release of pro-inflammatory mediators. While these biochemical reactions cause systemic alterations, the changes in the microvasculature also alter the amount of radiated heat from the body. With the use of an infrared camera, the coagulation zone with necrosis and lack of functional irrigation is detected at low temperatures. In contrast, vasoconstriction in the stasis zone and vasodilation in the hyperemia zone can be observed as yellowish-green and reddish color in the thermal images.

The World Health Organization reports a prevalence of 180,000 burn deaths each year, with a higher incidence in low- and middle-income countries, where non-fatal burns are one of the main causes of morbidity (93). Research into the pathophysiology of burns, burn wound healing, and projects designed to employ new treatment modalities constitute another aspect in which animal models are indispensable (94, 95). Thanks to these *in vivo* models, it has been possible to identify the inflammatory, circulatory, and metabolic alterations present in a burn injury, whether due to cold or heat (96). For example, in rodents, leporids, and pigs, the three characteristic regions of burn damage have been successfully reproduced: a central zone of necrosis or coagulation zone, a surrounding area of stasis with impeded blood flow, and an outermost zone of inflammation and hyperemia (Figure 6) (96, 97). According to the severity or extent of the damage, the classification of burns has changed from first, second, third, and fourth degree to represent the degree of burn depth as superficial, superficial partial-thickness, deep partial-thickness, and full-thickness (98).

The effects on dermal circulation differ according to the described regions. In the necrosis zone, there is a complete loss of dermis and subpapillary vasculature (96). In the stasis zone, there is no circulation from the dermal capillaries, whereas in the hyperemia zone, edema but intact vasculature making it recoverable tissue (96, 99). Because infrared thermography captures the R skin emissivity and the amount of heat radiated from the dermal blood vessels, IRT has been postulated as a complementary tool to analyze burn depth and healing potential (25). The visual technique for burn depth assessment has been shown to have only 60% accuracy (100). IRT has therefore been used to correlate temperature at the injury site with burn depth, as well as with progression and healing prediction (101).

### 7.1 Heat burn injury and IRT

Blood perfusion assessment helps predict healing time in burn patients, so techniques such as active dynamic thermography (ADT) can distinguish abnormalities in the heat transfer pattern of injured tissue. This was studied by Prindeze et al. (44) in a Duroc pig model with superficial partial and deep partial thickness burns. ADT identified different average temperatures from 30 to 150 min postinjury, an effect that was related to differences in perfusion (assessed with the LDI) between shallow and deep burns. In a porcine model using Yorkshire animals, IRT was applied, in conjunction with a multiprobe adapter system for transepidermal assessment and histology to determine burn severity over 4 days. The authors found that surface temperature decreased according to burn severity, being most notable in individuals with third-degree burns (baseline values of 34.4°C to a minimum of approximately 32°C in deep burns). This was consistent with increased transepidermal evaporative water loss and histological changes characteristic of deep burns (collagen coagulation, apoptosis, necrosis, and vascular occlusion). These results suggest that IRT is a tool that can establish burn severity (102).

In the same animal model with Yorkshire pigs, a burn depth assessment was also been performed by Ponticorvo et al. (103), who adopted the IRT method to assess burn severity. IRT was used as a noninvasive assessment method during the first 72 h to be compared with histological severity diagnosis. The results showed that IRT had an accuracy rate of 73% (correctly classifying 35 of 48 lesions), a sensitivity of 66%, and a specificity of 33%, in contrast to clinical assessment by histology, which had an accuracy of 83% (correctly classifying 40/48 burns). This suggests that, although IRT is a tool that can be adopted, a complementary assessment is required to correctly classify the severity of the lesions.

In third-degree burns, where the tissue is nonviable and does not heal, surgical excision and grafting are the appropriate options. However, because removal of viable tissue or incomplete removal of all necrotic tissue is common, it is essential to evaluate the healing process (102). In a comparative study in humans with third-degree burns between IRT and indocyanine green angiography (ICG), IRT was found to be able to determine the area of unsalvageable tissue, which overlapped 91% (range 82 to 98%) with the results obtained by ICG. However, because IRT overestimated the wound surface area by approximately 1 to 2 cm, it is important to establish that IRT can be applied in burns but must be accompanied by another diagnostic method (101). Similarly, although studies are carried out in animal models, clinical applications in human patients have shown that the temperature of burns classified microscopically as nondeep burns increase their temperature by an average of 1.5 ± 2.3°C from day 1 to 2 while in those classified as deep decreases of −1.5 ± 2.0°C were recorded (from 32.3 ± 2.0°C to 30.8 ± 1.3°C), keeping an accuracy of 87.2%, unlike the clinical assessment with 54.1% accuracy, which could help determine cases that require surgery (104).

### 7.2 Thermal imaging applied ice burns

In the case of frostbites, minipig models have shown changes in perfusion before and after frostbites, as reported by Rothenberger et al. (105). The authors recorded increases of 15–20% in blood flow for superficial and superficial partial burns, whereas in deep partial and full thickness burns blood flow decreased by 4 ± 2.1% to 27 ± 11.8%. These changes have been replicated in murine models and evaluated with IRT. Himashree et al. (106) used albino rats to induce superficial or deep frostbite burns on the pelvic limbs. Assessment during the first week showed that IRT detected areas of tissue viability and temperature increases in reversible (superficial) burns (from 17.6 ± 0.1°C immediately after frostbite to 23.1 ± 0.3°C) after 1 week of assessment. In contrast, lower values were recorded in deep (irreversible) burns than in superficial burns, and there was no significant increase in temperature (from 16.9 ± 0.2°C at 1 h post-injury to 17.1 ± 0.2°C 1 week later). Due to the nature of burns, the current assessment of burn depth is performed not only with IRT (due to its limitations) but with other methods such as Doppler laser, dermoscopy techniques, and hyperspectral imaging, among others (98).

## 8 Flaps and grafts, blood flow reconstruction that can be assessed through IRT

IRT can be used as an objective technique to monitor blood flow restoration after grafts and/or flaps (107). In grafts, IRT is ideal for identifying the thermal response derived from the formation of granulation tissue at the level of the dermis and epidermis, as well as from angiogenesis between the graft and the recipient area. Grafts are classified according to their origin. If they come from the same individual, they are known as autografts or isografts; if they come from a cadaveric donor, they are called allografts or homografts, and xenografts or heterografts if they come from a different species (108). Soft tissues such as skin, muscle, and fat may present alterations such as tumor masses, trauma, or congenital malformations that cause atrophy and dysfunction. Autologous free flap transplantation is used to repair such defects; however, it requires adequate blood perfusion and patency of the main artery in the anastomosis, and microcirculation of the transplanted tissue (13, 109).

In both grafts and flaps, IRT is an important tool that allows decisions to be made regarding the evolution of the procedure, especially considering that an undesirable ischemic process may be associated with a decrease in regional temperature or, on the contrary, an increase in temperature due to an inflammatory process, both affecting tissue integration. In an experimental surgical model of ischemia–reperfusion in flaps formed in rats, the importance of blood supply was analyzed by proposing the use of a hydrogel patch that releases carbon monoxide and nitric oxide gasses which favor blood supply to the flap, angiogenesis, and also promotes distal vascularization and inhibit the inflammatory process. Infrared thermal images obtained on day 14 post-surgery showed that the experimental group that received the hydrogel maintained a sustained blood supply compared to the other groups (109).

**Figure 7:**
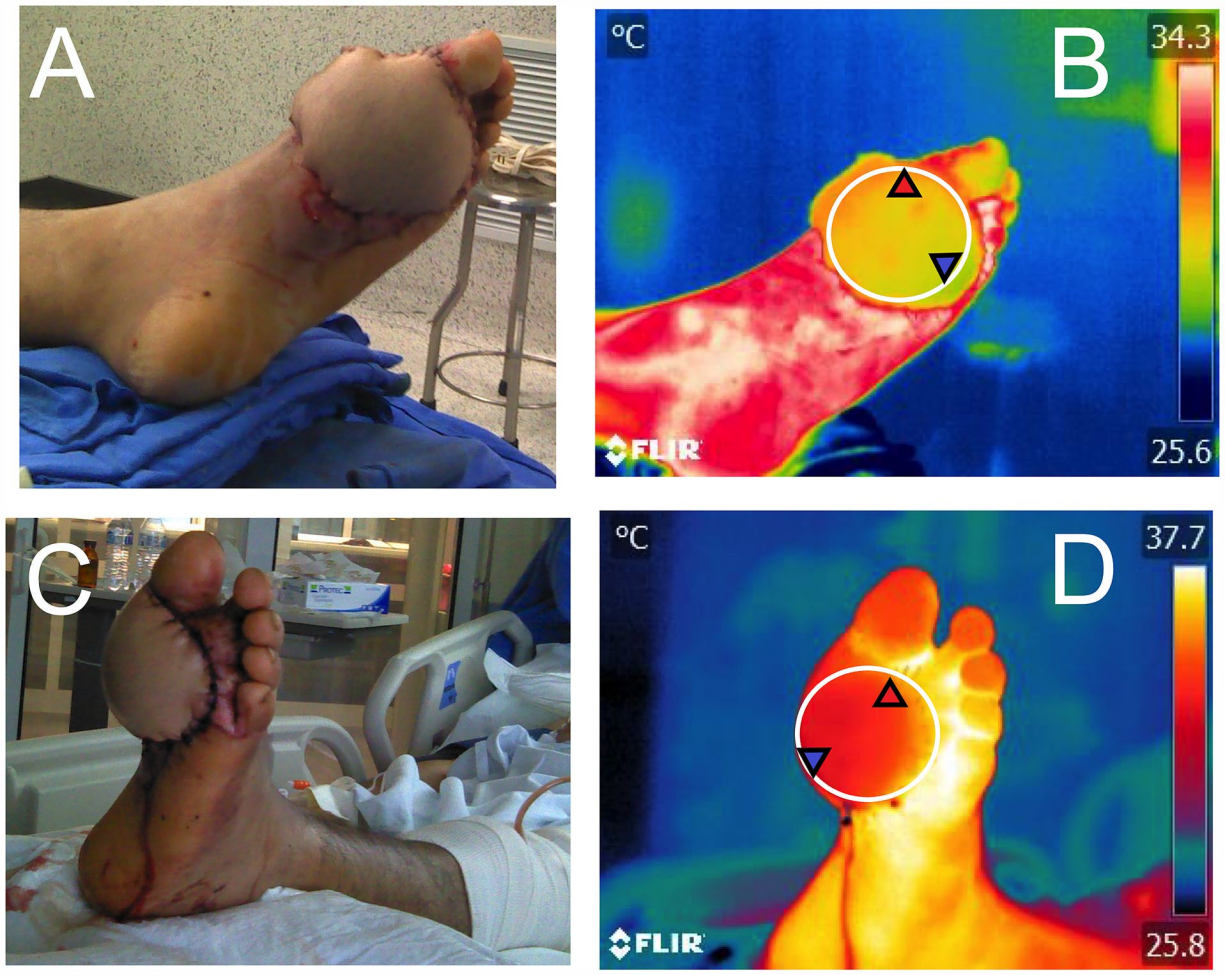
Use of infrared thermography to monitor peripheral irrigation in a flap. **(A)** and **(B)** immediate postoperative period after placement of a pedicled flap in a patient who received an electric shock and injured the medial plantar region of the left foot. The circle placed in Figure **B** (El1) shows the maximum (31.9°C), minimum (28.9°C), and average (30.1°C) temperature of the flap after the surgical procedure. Images **(C,D)** show the 24-h postoperative follow-up. Considering the circle that delimits the flap (El1) in Figure (D), it is observed that the maximum, minimum, and average temperatures increased by 3.7, 3.1, and 3.5°C, respectively, compared to the initial temperatures. One of the relevant clinical signs is the gradual increase in temperature in the flap region during the next hours, which is associated with blood flow and perfusion between the recipient tissue and the flap.

In an experimental model with rats, the role of defensins and the survival time of ischemic arterialized venous flaps intentionally infected with 10^5^ CFU of *Pseudomonas aeruginosa* was determined using IRT to confirm flap ischemia (i.e., decreased temperature in the left epigastric region of rats compared to its contralateral side) (110). In the same species, Czapla et al. (111) evaluated subcutaneous perforating arteries with IRT before experimental placement of the skin flap. The results showed that IRT identified suitable arteries with efficient blood flow for anastomosis, as well as visualizing those with a high probability of ischemia and necrosis. In humans, preliminary results of the present authors have shown that thermal imaging can be used to monitor flap viability, as shown in Figure 7.

## 9 From animal models to human medicine: translational use of IRT

The application of IRT in animal models has implications for both veterinary medicine and translational medicine. Although the use of alternatives to animal experimentation is currently advocated due to the differences that might be reported in physiological responses in animals and humans (112, 113), clinical trials developed in animals serve as an approximation to validate tools such as IRT. One of the most reported examples is studies evaluating tissue viability in patients who have received flaps after thermal or electrical burn wounds. In this regard, before performing graft or flap surgery, assessing burn wound depth using IRT has allowed for determining tissue healing potential with a sensitivity and specificity of 51.2 and 77.9% within the first 21 days in human patients (25). Higher percentages of sensitivity and specificity of IRT have been reported by Asif et al. (114) in humans.

These applications are relevant because early assessment of burn tissue viability might guide more accurate treatment protocols (e.g., flaps or grafts) (115). This has been reported in patients undergoing abdominoplasty, in whom alterations in blood perfusion of the transverse abdominal muscle showed differences in thermal patterns within 30 s. At a clinical level, the location of hot spots in the muscle can serve as a guide for the surgeon during the surgical procedure and in the recovery period. Similarly, the feasibility of hand reconstructions after traumatic hand injury has used dynamic infrared thermography during the postoperative period to continuously assess perforator flap perfusion (116).

**Figure 8:**
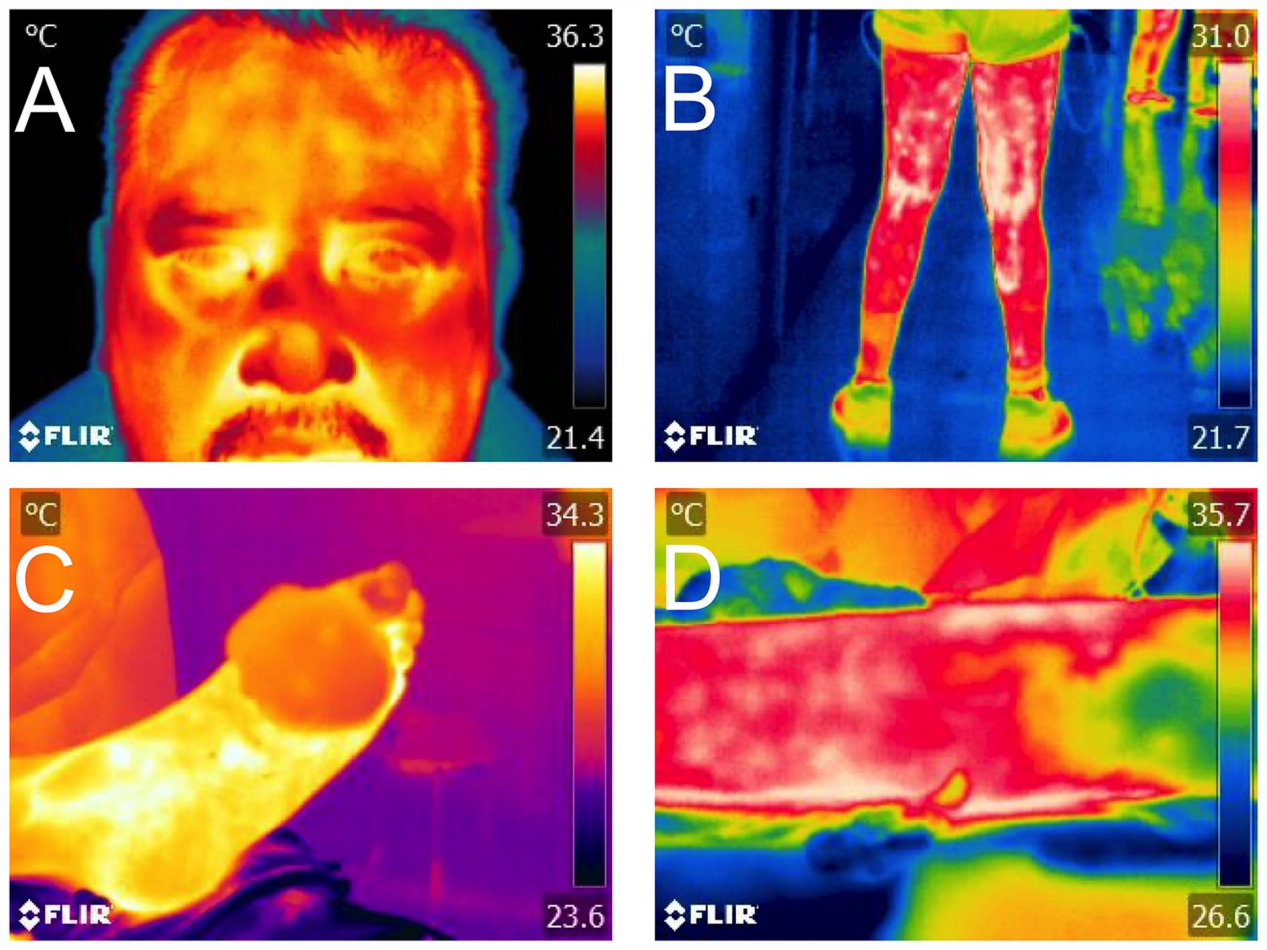
Application of IRT in both animal and human patients for translational medicine. **(A)** In patients with facial paralysis, temperature may be altered according to the vascular theory of the disease. **(B)** In rehabilitation medicine, restriction tapes are used to inhibit and promote blood return, aspects that can be measured with IRT. **(C)** In anastomosis surgery, indirect evaluation of revascularization through IRT in the postoperative period is a complementary tool to clinical tests. **(D)** In a burn injury, the extension of the affected area can be objectively visualized with IRT.

Burn injuries are the fourth most common type of trauma worldwide, causing significant morbidity and permanent disability, impacting the quality of life of the burned patient (117). The Luis Guillermo Ibarra-Ibarra National Rehabilitation Institute has a highly specialized center for the care of patients with severe burns, the CENIAQ (National Burn Research Center). In this center, protocols are being developed using IRT to evaluate the progression and treatment of patients with burn injuries. Moreover, other medical specialties linked to patient rehabilitation and physiotherapy have suggested IRT as a valuable tool for monitoring patient healing processes (Figure 8).

Another field in which animal models have served as an approximation to the expected results in humans is induced head trauma or neurosurgery and its association with thermal response. Cerebral vascular surgeries due to aneurysms have been studied with IRT. In these studies, by performing clip placement in the anterior cerebral artery, brain temperature before and after the procedure can be compared. Potential ischemic events were monitored through thermography, and the diagnosis provided by IRT was corroborated with computed tomography (CT) and angio-CT (118). Thus, IRT is currently used in various fields of human and animal medicine, and through studies carried out on animals, the basis for its application in the daily clinical practice of human patients can be obtained (13, 14, 119).

## 10 Limitations of thermal imaging that need to be considered by researchers

Several environmental, technical, and biological factors need to be considered when using thermal imaging due to its influence on the increases or decreases in surface temperature. Environmental conditions include direct solar radiation, wind speed, and relative humidity (120–122). For example, a linear correlation between surface temperature and ambient temperature was reported in pigs (slope value = 0.40°C) (123). Similarly, in horses, Soroko et al. (124) reported that increases in ambient temperature highly influence the surface temperature of the carpus region. Likewise, in rabbits, de Lima et al. (125) reported that ocular surface temperature is affected by temperature, humidity, and ventilation. The presence of wind and wind speed is another factor that reduces the surface temperature (126, 127). Wind speeds of 7 and 12 km/h reduce the ocular surface temperature by 0.43 and 0.78°C, respectively, in cattle (125, 127). Thus, the room where the animals are maintained needs to be within the animal’s thermal comfort zone and must be controlled to avoid inaccurate readings, as stated in dogs, in whom a controlled temperature of 21°C is recommended to reduce variations on IRT values (128).

Regarding technical factors or those related to the camera, the most critical aspect is related to emissivity. Emissivity is known as the object’s ability to emit radiation (129, 130). This ability depends on the characteristics of the surface or the skin. Animal models for biomedicine use a wide range of species with several traits such as the presence of hair, hair color, feathers, or glabrous skin that directly influence emissivity. For example, pigs have an emissivity of 0.92–0.95 (131), while horses and rabbits have recorded values of 0.95 and 0.97, respectively (132, 133). However, the selection of the thermal window is related to emissivity, as values can differ according to anatomical region, as shown in pigs, in whom the highest emissivity values were recorded at dorsal areas of live animals (0.93), and the lowest at hairy skin areas (0.93) or dead pig’s skin (0.80) (131). The differences in emissivity according to the species must be considered when using IRT in animal models that replicate human diseases, particularly when human skin emissivity is around 0.98 (131).

Another variable is related to the resolution of the camera, which might affect the quality of the data. In animals, a resolution between 80 × 90 pixels or 320 × 240 pixels is recommended (36, 134, 135), a resolution similar to the one is used in human studies (320 × 240 pixels). Additionally, the distance to the object and the capture angle must be considered to avoid recording inaccurate temperatures (136).

Biological aspects such as the color of the coat, hair length/type, dermal thickness, glabrous skin, and physiological differences (e.g., Wistar rats vs. naked mole rats) must be considered when selecting the animal model (137, 138). For example, in animals with hair, hair acts as a thermal insulator that affects infrared readings, decreasing the actual surface temperature (139). Thus, selecting the appropriate anatomical region to evaluate (e.g., zones without hair) and maintaining a controlled environment during experimental procedures is important when adopting IRT in research.

Finally, the use of IRT with other imaging techniques has been shown to improve the performance of thermal readings. An example is a study combining infrared thermography with laser speckle imaging to evaluate cerebral blood flow in rats as a model for stroke (60). In a model of peripheral arterial occlusion in Wistar rats, Hoinoiu et al. (140) evaluated tissue perfusion by laser Doppler and computed tomography angiography, where the thermographic analysis confirmed severe persistent ischemia. The importance of combining different diagnostic and monitoring methods is mentioned in a study where vascularization of induced mammary tumors in female Sprague–Dawley rats was performed through power Doppler, ultrasound (B flow), thermography, and histology analysis (141). In this study, the authors concluded that thermal monitoring was strongly correlated with Power Doppler, which might reflect the angiogenesis process during tumoral growth. Thus, in further animal models where IRT can be used, it is recommended to compare its application with other techniques.

## 11 Conclusion

Thermal imaging in biomedical animal models has been used to non-invasively detect temperature changes in blood vessels due to several events that alter blood flow. In real-time, IRT identifies increases or decreases in radiated heat according to blood circulation and microcirculation. Thus, IRT provides an alternative to evaluate vascular anomalies where blood flow is interrupted, resulting in reduced blood flow and radiated heat. In surgical processes such as anastomosis and reconstructive techniques (e.g., grafts and flaps), thermal imaging can assess the viability of tissues through blood flow restoration. Similarly, in burn injuries, IRT can predict the degree of tissular damage by delimiting the areas of ischemia-necrosis and inflammation. It is recommended to use IRT together with other diagnostic and imaging tools to provide an accurate diagnosis. Moreover, the application of IRT in several animal models could help to link this basic experimental information to its application in translational medicine.
